
JOURNAL INFORMATION
==============================
NLM Title Abbreviation: Am J Trop Med Hyg
Journal ID: Am J Trop Med Hyg
NLM Journal ID: 0370507
Journal ID: tpmd

The American Journal of Tropical Medicine and Hygiene

ISSN: 0002-9637
EISSN: 1476-1645
Publisher: The American Society of Tropical Medicine and Hygiene

ARTICLE INFORMATION
==============================
PMCID: PMC11965720
DOI: 10.4269/ajtmh.1124stoc
Article ID: tpmd1124stoc
Article version: 1
Subjects: Other

Contents

Print publication date: 2025 Apr
Electronic publication date: 2025 Apr 1
Volume: 112
Issue: 4 Suppl
Issue title: Rapid Operational Research of SARS-CoV-2 Rapid Tests to Combat COVID-19 in Communities
First page: ii
Last page: iii
Copyright: © The American Society of Tropical Medicine and Hygiene
Copyright year: 2025
License: This is an open-access article distributed under the terms of the Creative Commons Attribution (CC-BY) License, which permits unrestricted use, distribution, and reproduction in any medium, provided the original author and source are credited.
License URL: https://creativecommons.org/licenses/by/4.0/

Page count: 2

==============================

### Rapid Operational Research of SARS-CoV-2 Rapid Tests to Combat COVID-19 in Communities

### Guest Editors: Daniel G. Bausch and Ezekiel Boro

### CONTENTS

**EDITORIAL**

**Advancing Global Health Security through Rapid Operational Research on Diagnostics during Outbreaks**
Ezekiel Boro, Anne Hoppe, and Daniel G. Bausch 1

**ARTICLES**

**Successful Integration of Community-Based Rapid Antigen Testing for COVID-19 and Malaria in Mali**
Guillaume Breton, Issouf Maïga, Aboubacar Maïga, Gabrièle Laborde Balen, Luis Sagaon-Teyssier, Anne Hoppe, Odé Kanku Kabemba, Boubacar Cissé, and Fatou Diawara Traore 3

**Uptake, Acceptability, and Results of SARS-CoV-2 Antigen Rapid Diagnostic Testing in Community Settings in Cameroon**
Tatiana K. Djikeussi, Boris Kevin Tchounga, Loic Feuzeu, Rogacien Kana, Boris Tchakounte Youngui, Shannon Viana, Heather J. Hoffman, Albert Mambo, Charlotte Moussi, Joseph Fokam, Emilienne Epée, Anne Hoppe, Pallavi Dani, Patrice Tchendjou, Laura Guay, and Michelle M. Gill 10

**A Cross-Sectional Study of the Use of Antigen Rapid Diagnostic Tests for Community Identification of SARS-CoV-2 in Kenya**
Rose Masaba, Stephen Siamba, Heather J. Hoffman, Edyth Osire, Njoki Kimani, Magoma Kwasa, Mario Songane, and Lise Denoeud-Ndam 17

**Acceptability, Feasibility, and Uptake of COVID-19 Antigen Rapid Diagnostic Self-Testing at the Community Level in Tanzania**
Grace W. Mwangoka, Ali M. Ali, Mwifadhi Mrisho, Abdallah Mkopi, Muhidin Mahende, Hajirani M. Msuya, Silas G. Temu, Paul Kazyoba, Michael G. Mihayo, Omar Juma, Ali Hamad, Said A. Jongo, Omar Lweno, Anneth Tumbo, Sara S. Mswata, Anne Hoppe, Pallavi Dani, and Salim Abdulla 26

**Sociodemographic and Psychosocial Factors Influencing COVID-19 Testing Uptake: Insights from Urban and Rural Communities in South Africa**
Nokhanyo Xaba, Onaiza Qureshi, Aneeta Pasha, Amyn Malik, Anne Hoppe, Zaw Myo Tun, National Fynn, Goodman Sibeko, Saira Khowaja, and Aamir Javed Khan 37

**Feasibility and Effectiveness of Using Community Testing Centers to Increase Access to COVID-19 Testing Services in Urban Mozambique**
Júlia Sambo, Nádia Sitoe, Neuza Nguenha, Jorfélia Chilaúle, Phath Guambe, Denise Langa, Júlio Rafael, Chishamiso Mudenyanga, Nédio Mabunda, Osvaldo Loquiha, and Ilesh Jani 46

**Consecutive Evaluation of Systematic Community-Based COVID-19 Antigen Rapid Diagnostic Testing in Three Different Populations in Jamaica during the COVID-19 Pandemic**
David Walcott, Chika Ozongwu, Carl Bruce, Alison Nicholson, Camille-Ann Thoms Rodriguez, Jerome Patrick Walker, John Lindo, Melanie Dawkins, Samantha Johnson, Kristen Collins, Tresana Pearson, Vanessa Bailey-Higgins, Pallavi Dani, Shaquielle Dias, and Anne Hoppe 53

**Community-Based Delivery and Administration of SARS-CoV-2 Antigen Rapid Diagnostic Tests: An Operational Research Study in Marketplaces in Malawi and Zambia**
Fiona Gambanga, Lindiwe Nchimunya, Joseph Makondesa, Chancy Chavula, Namwaka Mulenga, Tamara Mwenifumbo, Francis Chitanda, Jonathan Mtaula, Yucheng Tsai, Joseph Bitilinyu-Bangoh, Andrews Gunda, Aaron Shibemba, Powell Choonga, Shaukat Khan, and Trevor Peter 63

**Optimizing the Use of SARS-CoV-2 Antigen Rapid Diagnostic Tests for the Timely Detection of and Response to COVID-19 in Schools and Markets in Uganda**
Jerry Mulondo, Susan Nayiga, Winnie Nuwagaba, Patience Nayebare, Jane Frances Namuganga, Isaac Ssewanyana, Moses R. Kamya, and Joaniter I. Nankabirwa 71

**Feasibility and Acceptability of SARS-CoV-2 Antigen Rapid Diagnostic Testing in High-Risk Markets and Trade Hubs in Kampala, Uganda**
Isaac Ssewanyana, Sam Acellam, Martha Ampumuza, Hellen Nansumba, Susan Nabadda, Grace Kushemererwa, Victor Bigira, Sarah Zalwango, Chris Oundo, Richard Walyomo, Isaiah Chebrot, Dennis Mike Buluma, Alex Ndyabakira, Pallavi Dani, and Anne Hoppe 79

**Use of Antigen Rapid Diagnostic Test for Detection of COVID-19 Cases in University Settings in Cameroon**
Chavely Gwladys Monamele, Landry Messanga, Nicolas Njintang Yanou, Gustave Simo, Carole Else Eboumbou Moukoko, Henri Moumbeket, Abdou Fatawou Modyinyi, Ripa Mohamadou, Yacouba Foupouapouognigni, Armand Abdou, Jane-Francis Tatah Kihla Akoachere, Pallavi Dani, Anne Hoppe, and Richard Njouom 84

**Assessing the Benefit of Two Detection Approaches in Screening COVID-19 Using SARS-CoV-2 Antigen Rapid Diagnostic Tests among Intercity Public Bus Travelers in Cameroon**
Jerome Ateudjieu, Ketina Hirma Tchio-Nighie, Anne Hoppe, Etienne Guenou, Imelda Sonia Nzinnou Mbiaketcha, Landry Beyala Bita'a, Claudine Sen Henriette Ngomtcho, and Anne Cecile Bissek 92

For more information about
the American Society of Tropical Medicine and Hygiene
please visit us at www.astmh.org

The American Journal of Tropical Medicine and Hygiene is
online at www.ajtmh.org

**Implementing SARS-CoV-2 Testing during a Large-Scale Sporting Event in Africa: Lessons Learned from the Africa Football Cup of Nations Tournament in Cameroon**
Boris K. Tchounga, Boris Tchakounte Youngui, Emilienne Epée, Tatiana Djikeussi, Joseph Fokam, André P. Goura, Loic Feuzeu, Muhamed Awulo Mbunka, Pallavi Dani, Shannon Viana, Anne Hoppe, Yap Boum, Rhoderick Machekano, Laura Guay, Anne-Cecile Zoung-Kanyi Bissek, John Ditekemena, Appolinaire Tiam, Alain G. Etoundi, Patrice Tchendjou, and Michelle M. Gill **99**

**Acceptability and Feasibility of Provision of COVID-19 Services by Community Health Workers to Remote Gold Mining Communities in Suriname**
Stephen Vreden, Marieke Heemskerk, Hélène Hiwat, and Hedley Cairo **109**

**Operational Research to Support Rapid Evidence-Based Responses to Outbreaks: Learnings from COVID-19**
Anne Hoppe, Pallavi Dani, Grace Mwangoka, Stephen Vreden, Guillaume Breton, Jerome Ateudjieu, Joaniter I. Nankabirwa, Júlia Sambo, Rose Masaba, Tatenda Maparo, Goodman Sibeko, Richard Njouom, Boris Tchounga, Isaac Ssewanyana, Chancy Chavula, Lindiwe Nchimunya, Tatiana Djikeussi, Sam Accellam, Hedley Cairo, David Walcott, Aamir J. Khan, Shaukat Khan, and Daniel G. Bausch **119**
